# IgG Binds *Escherichia coli* Serine Protease EspP and Protects Mice From *E. coli* O157:H7 Infection

**DOI:** 10.3389/fimmu.2022.807959

**Published:** 2022-02-18

**Authors:** Ashmita Tontanahal, Vanessa Sperandio, Olga Kovbasnjuk, Sebastian Loos, Ann-Charlotte Kristoffersson, Diana Karpman, Ida Arvidsson

**Affiliations:** ^1^ Department of Pediatrics, Clinical Sciences Lund, Lund University, Lund, Sweden; ^2^ Departments of Microbiology and Biochemistry, University of Texas Southwestern Medical Center, Dallas, TX, United States; ^3^ Division of Gastroenterology, Department of Internal Medicine, University of New Mexico Health Science Center, Albuquerque, NM, United States

**Keywords:** *Escherichia coli* O157:H7, Shiga toxin, EspP, immunoglobulin G, hemolytic uremic syndrome, mouse

## Abstract

Shiga toxin-producing *Escherichia coli* O157:H7 is a virulent strain causing severe gastrointestinal infection, hemolytic uremic syndrome and death. To date there are no specific therapies to reduce progression of disease. Here we investigated the effect of pooled immunoglobulins (IgG) on the course of disease in a mouse model of intragastric *E. coli* O157:H7 inoculation. Intraperitoneal administration of murine IgG on day 3, or both on day 3 and 6, post-inoculation improved survival and decreased intestinal and renal pathology. When given on both day 3 and 6 post-inoculation IgG treatment also improved kidney function in infected mice. Murine and human commercially available IgG preparations bound to proteins in culture filtrates from *E. coli* O157:H7. Bound proteins were extracted from membranes and peptide sequences were identified by mass spectrometry. The findings showed that murine and human IgG bound to *E. coli* extracellular serine protease P (EspP) in the culture filtrate, *via* the IgG Fc domain. These results were confirmed using purified recombinant EspP and comparing culture filtrates from the wild-type *E. coli* O157:H7 strain to a deletion mutant lacking *espP.* Culture filtrates from wild-type *E. coli* O157:H7 exhibited enzymatic activity, specifically associated with the presence of EspP and demonstrated as pepsin cleavage, which was reduced in the presence of murine and human IgG. EspP is a virulence factor previously shown to promote colonic cell injury and the uptake of Shiga toxin by intestinal cells. The results presented here suggest that IgG binds to EspP, blocks its enzymatic activity, and protects the host from *E. coli* O157:H7 infection, even when given post-inoculation.

## Introduction

Enterohemorrhagic *Escherichia coli* (EHEC) is a human pathogen, transmitted *via* contaminated food and water causing diarrhea and hemorrhagic colitis. It is the main cause of hemolytic uremic syndrome (HUS) (1). EHEC is a non-invasive bacterium (2) that exerts its effects by the release of virulence factors such as Shiga toxin (3, 4). The most common clinical isolate is *E. coli* O157:H7 (5). Upon ingestion, EHEC is transported to the large intestine where it colonizes the gut by intimate attachment to intestinal epithelial cells, leading to formation of attaching and effacing (A/E) lesions mediated by a type III secretion system (T3SS), intimin, translocated intimin receptor and *E. coli* secreted proteins (6, 7).

Release of *E. coli* secreted proteins by the bacteria is essential for formation of A/E lesions in the host cells (8). However, even T3SS-negative strains can induce diarrhea (9). Diarrheagenic *E. coli* secrete serine proteases by means of a type V secretion system, the Serine Protease Autotransporter of Enterobacteriaceae (SPATEs) protein family (10). These proteases function as enterotoxins thereby causing diarrhea (11). One such protease in EHEC is extracellular serine protease P (EspP) shown to be important for adherence to bovine intestinal cells (12) and ion transport in human colonoid cells, that could suggest a role in the development of watery diarrhea (11). The presence of EspP was associated with highly pathogenic EHEC strains (13). EspP cleaves coagulation factor V (14) and complement C3, C3b and C5 (15) showing that it could impact host proteins important for coagulation and complement activation.

To this date there are no effective treatments for EHEC infection. Antibodies that target specific virulence factors could be an attractive option, such as antibodies against Shiga toxin (16), or components of the T3SS (17), that are under development (18). Immunoglobulin Y (from egg yolk) anti-*E. coli* O157 was shown to inhibit growth of *E. coli* O157:H7 (19). Earlier studies investigated the effect of pooled immunoglobulin G (IgG) in patients with EHEC infections. Antibodies against Shiga toxin 1 were detected in IgG preparations (20), but clinically relevant bacterial isolates usually release Shiga toxin 2. Treatment of pediatric patients with EHEC-associated diarrhea with bovine colostrum concentrate (containing high levels of IgG) reduced the stool frequency (21). Administration of intravenous IgG to HUS patients exhibited equivocal results with a protective effect in one study (22) and no effect in another (23), which could be due to the timing of administration after the development of HUS.

In this study an established mouse model of intragastric *E. coli* O157:H7 infection (24) was used to study the effect of IgG on the course of disease. *In vitro* studies were conducted to assess the interaction between murine or human IgG preparations and *E. coli* O157:H7 proteins which led us to the finding that IgG binds specifically to EspP *via* its Fc domain, and neutralizes protease activity. IgG binding to EHEC EspP could explain the protective effect on the course of infection.

## Material and Methods

### Murine and Human Immunoglobulin Purification and Isotyping

Mouse IgG was purified from two separate batches of mouse sera (Sigma-Aldrich, Steinheim, Germany) using protein G sepharose (GE Healthcare, Uppsala, Sweden) and eluted with glycine buffer (0.1M, pH 2.5) followed by neutralization with TRIS-HCl (1M, pH 9). The immunoglobulin fraction was dialyzed against PBS overnight at 4°C, filtered (0.2 μm, Pall Corp., Ann Arbor, MI) and the amount of IgG was measured using a NanoDrop spectrophotometer (ND-1000, Saveen & Werner, Limhamn, Sweden). To isotype the immunoglobulin fraction, Pierce Rapid ELISA isotyping kit (Thermo Fisher Scientific, Waltham, MA) was used according to the manufacturer’s instructions. The sample consisted of more than 80% IgG kappa with IgG2b as the most abundant isotype.

Human IgG was used from a preparation of Privigen CLS Behring, Marburg, Germany (Lot: P100113155). The purity of Privigen is 98% consisting of IgG1 69%, IgG2 26%, IgG3 3% and IgG4 2% and a maximal amount of IgA 25 µg/mL according to the manufacturer.

Fab and Fc fragments were generated from mouse and human IgG using Pierce Fab preparation kit (Thermo Fisher Scientific) containing papain, according to the manufacturer’s instructions.

### Mice

Female and male BALB/c mice aged 8-12 weeks were bred and used for experiments at the Center for Comparative Medicine, Medical Faculty, Lund University. All animal experiments were approved (approval numbers M13-14 and M76-15) by the animal ethics committee of Lund University in accordance to the guidelines of the Swedish National Board of Agriculture and the EU directive for the protection of animals used in science.

### Bacterial Strains

The *Escherichia coli* O157:H7 strain (86-24) isolated from the Walla Walla outbreak in 1986 (25) was kindly provided by A.D. O’Brien (Department of Microbiology and Immunology, Uniformed Services University of the Health Sciences, Bethesda, MD). This wild-type strain was previously characterized (4) and a streptomycin-resistant (StrR) derivate was used (24). In addition, a previously described EspP deletion mutant, 86-24 Δ*espP* StrR (26), was used in certain experiments.

### Infection Protocol

BALB/c mice were treated with streptomycin 5 g/L in tap water from one day before inoculation and throughout the length of the experiment. Prior to inoculation, the mice were fasted for 16 h, for food but not water. Mice were anesthetized with isoflurane and inoculated intragastrically with 100 μl of *E. coli* O157:H7 (86-24 StrR, wild-type) bacterial suspension in 20% sucrose and 10% NaHCO_3_ at a final concentration of 10^9^ colony forming units/ml or vehicle alone as previously described (24) *via* a soft polyethylene catheter (Clay Adams, Parsippany, NJ). After inoculation food was reintroduced *ad libitum*.

Mice were monitored for 14 days with weight being recorded daily from one day before bacterial inoculation. Feces was collected on days 1, 3, 5 and 8 and serially diluted in PBS before plating on Luria Bertani (LB)-agar plates supplemented with streptomycin (50 μg/mL). Colonies were confirmed to be *E. coli* O157:H7 using an *E. coli* O157:H7 latex kit (Oxoid, Basingstoke). Mice were monitored for symptoms such as decreased activity, hunched posture, tremors, stillness and/or weight loss of ≥ 20%. Upon development of one of these symptoms, mice were sacrificed, while unaffected mice were sacrificed at the end of the experiment.

### Intraperitoneal Injection of Mouse IgG

BALB/c mice (infected intragastrically with *E. coli* O157:H7 or given vehicle alone) were injected intraperitoneally (i.p.) with protein G purified mouse IgG (1 mg/mouse in 1 ml PBS, or 1 ml PBS alone) on day 3, or on day 6, or on both day 3 and 6 post-inoculation with *E. coli* O157:H7.

### Blood Collection and Measurement of Blood Urea Nitrogen

Blood was collected in EDTA (0.1 M, Merck, Darmstadt, Germany) at the end of the experiment under anesthesia (Isoflurane, Forene Abbott, Wiesbaden, Germany) *via* heart puncture. Blood was centrifuged at 1500 x g for 15 min and 13000 x g for 3 min at room temperature (RT) and stored at -80°C for up to 4 months until assayed. Plasma samples were used to measure blood urea nitrogen using QuantiChrom Urea assay kit (BioAssay systems, Hayward, CA) according to the manufacturer’s instructions.

### Histopathological Analysis

Intestines and kidneys were removed after sacrifice when mice developed symptoms or, if unaffected, at the end of the experiment (day 14). The tissues were fixed in paraformaldehyde (4%, Histolab, Gothenburg, Sweden), embedded in paraffin, sectioned (3 µm) and stained with hematoxylin and eosin. All sections were coded and analyzed in a blinded manner. Images were obtained using Nikon TiEclipse microscope equipped with Nikon color camera (Nikon Instruments Inc., Tokyo, Japan) and analyzed with NIS elements AR software v.5.11.01. Sections of the entire colon and kidney were analyzed for pathology, specifically inflammatory infiltrates and goblet cell depletion in the colons and tubular epithelial desquamation in the kidneys. Pathology in intestines and kidneys was graded as: 0: not observed, 1: mild, 2: moderate and 3: severe.

### Bacterial Culture Filtrates

*E. coli* O157:H7 strains 86-24 wild-type StrR and 86-24 Δ*espP* StrR were cultured in LB broth for 24 h at 37°C. The bacterial suspension was centrifuged at 1500 x g for 10 min, the supernatant was transferred to a new tube, centrifuged at 13000 x g for 3 min at RT, filtered (0.2 μm) and stored at -80°C until used. The culture filtrate was used for detection of IgG binding to *E. coli* O157:H7 proteins and for assay of EspP enzymatic activity.

### Detection of IgG Binding to *Escherichia coli* O157:H7 Proteins and Lipopolysaccharide by Enzyme-Linked Immunosorbent Assay

IgG binding to *E. coli* O157:H7 proteins and lipopolysaccharide was detected by enzyme-linked immunosorbent assay. To detect IgG binding to *E. coli* O157:H7 proteins, *E. coli* O157:H7 culture filtrates (concentration 10, 5, 2.5 and 1.25%), Shiga toxin 2 (1 μg/mL, Phoenix Lab, Tufts Medical Center, Boston, MA) or O157LPS (1 μg/mL, a gift from R. Johnson, Public Health Agency, Guelph, ON, Canada), diluted in PBS (pH 7.4) were coated on a white 96-well Maxisorp plate (Nunc, Roskilde, Denmark) overnight at 4°C. The plate was washed three times with PBS-Tween (Medicago, Uppsala, Sweden) and blocked with 1% bovine serum albumin (BSA) (Sigma-Aldrich) in PBS. For antibody binding to *E. coli* O157:H7 proteins, purified mouse or human IgG (described above, 1 μg/mL diluted in 1% BSA) or 1% BSA (negative control), were added to the plate and incubated for 1h at RT. The plate was washed as above and incubated for 1 h at RT with goat anti-mouse IgG HRP (1:1000, Dako, Glostrup, Denmark) or rabbit anti-human IgG HRP (1:1000, Dako). The plate was washed as above and IgG binding was detected using Super signal ELISA pico chemiluminescent substrate (Thermo Fisher Scientific) and measured using Glomax Discovery system (Promega, Madison, WI).

### Detection of IgG Binding to *Escherichia coli* O157:H7 Proteins by Immunoblotting

*E. coli* O157:H7 culture filtrates from strains 86-24 wild-type StrR and 86-24 Δ*espP* StrR were concentrated 30x using Amicon^®^ centrifugal filter 10 kDa cutoff (Sigma-Aldrich). The concentrate (10 μl diluted 1:2 in sample buffer) or purified EspP (2 µg) (11) were run unreduced on a 4-20% TGX gel (Bio-Rad) and transferred to a polyvinylidene difluoride (PVDF) membrane (Bio-Rad). The membrane was blocked with 1x casein (Vector Laboratories, Burlingame, CA) overnight at 4°C, followed by incubation with mouse IgG or human IgG (1 μg/mL) or the corresponding Fab or Fc fragments (also at 1 μg/mL) for 1 h at RT. The membrane was washed three times with PBS-Tween, incubated with goat anti-mouse IgG HRP (1:1000) or rabbit anti-human IgG HRP (1:1000) for 1 h at RT and bound proteins were visualized using ECL Plus (Thermo Fisher Scientific).

### Isolation of IgG-Binding *E. coli* O157:H7 Proteins

PVDF membranes containing *E. coli* O157:H7 proteins were cut into segments based on the molecular weight of the different bands detected by mouse or human IgG. The proteins were eluted from the membrane using 1% Triton X-100/20% acetonitrile in 50 mM Tris-HCl (all from Sigma-Aldrich), pH 9.0, as previously described (27). To remove the detergent from the samples HiPPR detergent removal spin columns (Thermo Fisher Scientific) were used according to the manufacturer’s instructions. To confirm that proteins were purified, eluted samples were run on gels as described above and stained using Pierce silver stain kit (Thermo Fisher Scientific) according to the manufacturer’s instructions or transferred to a PVDF membrane and incubated with mouse or human IgG as described above.

### Mass Spectrometry Sample Preparation

Membrane eluted proteins were reduced with 10 mM dithiothreitol for 30 min at 56°C and alkylated with 20 mM iodoacetamide for 30 min at RT in the dark. The samples were precipitated overnight at -80°C using ethanol 99.5%. Precipitated samples were centrifuged at 14000 x g for 15 min at 4°C, the pellet was dissolved in 25mM ammonium bicarbonate and digested to peptides using trypsin (Sigma-Aldrich) for 18 h at 37°C. The reaction was stopped using 5 μl of 10% trifluoroacetic acid (Sigma-Aldrich), desalted using Ultra Microspin C18 columns (Nest Group, Southborough, MA) and dried using a SpeedVac. The dried peptides were dissolved in 25 µl of 2% acetonitrile, 0.1% trifluoroacetic acid (Sigma-Aldrich) and the concentration was measured using a DS-11 FX spectrophotometer (DENovix Inc, Wilmington, DE).

### Mass Spectrometry Acquisition

Liquid chromatography-mass spectrometry (LC-MS/MS) detection was performed to identify proteins or peptides from *E. coli* O157:H7 culture filtrates that interacted with IgG. LC-MS/MS detection was performed on a Tribrid mass spectrometer Fusion equipped with a Nanospray Flex ion source and coupled with an EASY-nLC 1000 ultrahigh pressure liquid chromatography (UHPLC) pump (Thermo Fisher Scientific). Peptides (prepared as described above, 1 µg) were injected into the LC-MS/MS. Following this the peptides were concentrated on an Acclaim PepMap 100 C18 precolumn (75 μm x 2 cm, Thermo Fisher Scientific) and then separated on an Acclaim PepMap RSLC column (75 μm x 25 cm, C18, 2 μm, 100 Å, nanoViper) at 40°C and a flow rate of 300 nL/min. Solvent A (0.1% formic acid in water) and solvent B (0.1% formic acid in acetonitrile) were used to create a nonlinear gradient to elute the peptides. For the gradient, the percentage of solvent B was maintained at 3% for 3 min, increased to 25% for 60 min, to 60% for 10 min and to 90% for 2 min after which it was kept at 90% for another 8 min to wash the column.

Orbitrap Fusion was operated in the positive data-dependent acquisition (DDA) mode. The separated peptides were ionized *via* stainless steel Nano-bore emitter (OD 150 µm, ID 30 µm) with a spray voltage of 2 kV and the capillary temperature was set at 275°C. Full MS survey scans from m/z 350-1350 with a resolution of 120,000 were performed in the Orbitrap detector. The automatic gain control (AGC) target was set to 4 × 10^5^ with an injection time of 50 ms. The most intense ions (up to 20) with charge states 2-5 from the full scan MS were selected for fragmentation in the Orbitrap. The precursors in the second analyzer were isolated with a quadrupole mass filter set to a width of 1.2 m/z. Precursors were fragmented by high-energy collision dissociation (HCD) at a normalized collision energy (NCE) of 30%. The resolution was fixed at 30,000 and for the MS/MS scans, the values for the AGC target and injection time were 5 × 10^4^ and 54 ms, respectively. The duration of dynamic exclusion was set to 45s and the mass tolerance window was 10 ppm.

### Mass Spectrometry Analysis

The list of detected peptides/proteins was sorted based on size, number of peptides detected and peptide score, matching amino acid sequences to detect a protein, from which the peptides are derived, with higher certainty. Proteins with a lower molecular size, compared to the detected proteins in immunoblots, as well as all detected proteins with only one detected peptide were excluded. The list of proteins was ranked based on peptide score and analyzed for identification of redundancy in the different samples.

### EspP Enzymatic Activity

EspP has been previously shown to cleave pepsin (28). *E. coli* O157:H7 culture filtrates from strains 86-24 wild-type StrR and 86-24 Δ*espP* StrR were concentrated 100x using Amicon^®^ centrifugal filter 50 kDa cutoff (Sigma-Aldrich). Supernatants (5 µl) were preincubated with or without mouse IgG (5 µg), human IgG (100 µg) or as a control the serine protease inhibitor phenylmethylsulfonyl fluoride (PMSF) (1x, G-Biosciences, St Louis, MO) for 10 min at RT. Pepsin was added (6 µg, from pig gastric mucosa, Roche, Mannheim, Germany) and the samples (final volume 10 µl) were incubated for 16 h at 37°C. After incubation one microliter unreduced sample was loaded onto a 4-20% TGX gel (Bio-Rad), transferred to a PVDF (Bio-Rad) membrane, and blocked using 1x casein as described above. The membrane was incubated with polyclonal goat IgG anti-pepsin (1:5000, Abcam, Cambridge, UK) for 1 h at RT, washed with PBS-Tween, incubated with rabbit anti-goat IgG HRP (1:1000, Dako) and visualized using ECL Plus (Thermo Fisher Scientific).

### Statistics

Differences between two groups were analyzed by the two-tailed Mann-Whitney U-test and when comparing more than two groups the Kruskal-Wallis test followed by Dunn’s procedure was performed. Kaplan-Meier survival curves were analyzed using the log-rank test. All statistical analyses were performed using Prism 9 version 9.1.1 (GraphPad, La Jolla, CA) and P ≤ 0.05 was considered significant.

## Results

### Mouse IgG Protected Mice From *E. coli* O157:H7-Induced Disease

**Figure 1:**
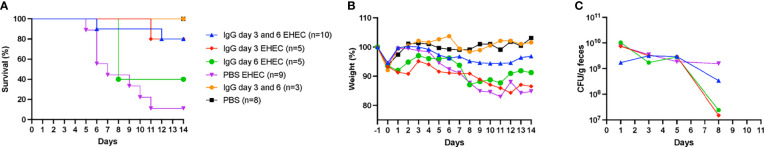
Murine IgG given on day 3 or day 3 and 6 protected mice from *E. coli* O157:H7-induced disease. **(A)** Survival in mice infected with *E. coli* O157:H7 (EHEC) and treated with IgG on day 3 and 6 (n=10, blue line), day 3 (n=5, red line), day 6 (n=5, green line), untreated (n=9, purple line) and uninfected controls treated with IgG (n=3, yellow line) and without IgG (n=8, black line). **(B)** Weight changes in infected and uninfected mice, starting 1 day before inoculation, at the start of fasting, until day 14 post-inoculation, when the experiment ended. **(C)** Bacterial colony forming units in feces of EHEC infected mice on days 1, 3, 5 and 8. Results are presented as medians.

Mice were inoculated orally with *E. coli* O157:H7 and treated intraperitoneally with IgG on day 3 and/or day 6 or left untreated for 14 days and sacrificed upon development of symptoms or at the end of the experiment. Infected and untreated mice started to develop symptoms on day 5 and by day 14 only 1/9 (11%) survived. Mice treated with IgG on days 3 and 6 started to develop symptoms on day 6 and by day 14, 8/10 of the mice survived, **Figure 1A**. A significant difference in survival was found between mice infected with *E. coli* O157:H7 and treated with IgG on day 3 and 6 compared to infected and untreated mice, P<0.01. Likewise, mice infected with *E. coli* O157:H7 and treated with IgG on day 3 post-inoculation exhibited 80% survival (4/5 mice), compared to infected and untreated mice, P<0.01. There was no significant difference in survival between mice infected with *E. coli* O157:H7 and treated with IgG on day 6 post-inoculation (40% survival, 2/5) compared to infected and untreated mice.

Mice infected with *E. coli* O157:H7 and IgG-treated or untreated were monitored for weight loss as shown in **Figure 1B**. Infected and untreated mice started to lose weight on day 5 and continuously lost weight until 8/9 mice were sacrificed by day 11. In contrast, mice infected and IgG-treated twice on day 3 and 6 demonstrated stable weight throughout the entire experiment whereas mice infected and IgG-treated once, on day 3 or day 6, demonstrated weight loss. Unexpectedly, mice in the group that was infected and IgG-treated on day 3 did not gain back their original weight (from before fasting) which was not observed for the other groups.

Fecal colony forming units were measured in infected mice on days 1, 3, 5 and 8, **Figure 1C**. A difference in colony forming units was observed on day 8 between infected and untreated mice (4/9 mice survived until this day) compared to mice infected and IgG-treated on day 3 (5/5 mice survived on day 8), P< 0.05. On day 8 there was no difference in colonization between the infected and untreated group and the other groups (IgG-treated twice on day 3 and day 6 in which 9/10 survived, as well as IgG-treated on day 6 in which 2/5 mice survived). The results suggest that IgG treatment had a marginal effect on colonization.

### Pathological Findings in IgG-Treated or Untreated Mice Infected With *E. coli* O157:H7

**Figure 2:**
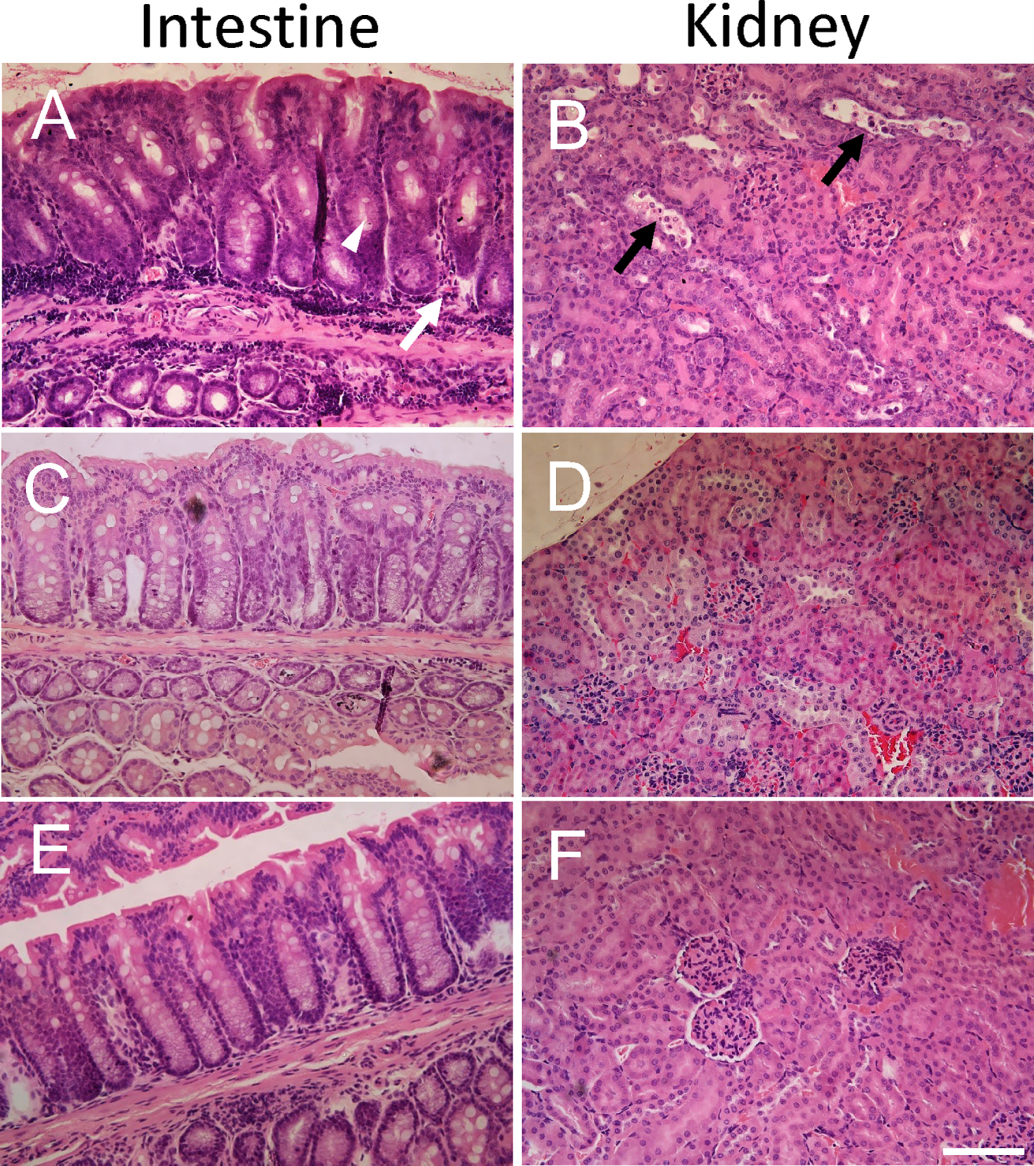
Histopathology in *E. coli* O157:H7-infected mice. Pathological changes were found in untreated *E. coli* O157:H7-infected mice. **(A)** Intestinal inflammatory infiltrates (white arrow) and mucus-depleted goblet cells (white arrowhead) in a mouse sacrificed on day 10 after inoculation in the infected and untreated group (corresponding to pathological score 2 for inflammatory infiltrates and pathological score 3 for mucus-depleted goblet cells). **(B)** Renal tubular desquamation (black arrow) in a mouse sacrificed on day 10 after inoculation in the infected and untreated group (corresponding to pathological score 3). **(C)** Normal intestinal histology in infected and IgG treated mouse on day 3 sacrificed on day 14 (corresponding to pathological score 0). **(D)** Normal renal histology in infected and IgG treated mouse on day 3 and 6 sacrificed on day 14 (corresponding to pathological score 0). **(E)** Normal intestinal histology in a control mouse sacrificed on day 14 (corresponding to pathological score 0). **(F)** Normal renal histology in a control mouse sacrificed on day 14 (corresponding to pathological score 0). Scale bar 100 μm.

Intestines were scored in blinded fashion for the degree of intestinal inflammatory infiltrates and goblet cell depletion and kidneys for renal tubular epithelial desquamation. Pathological findings in mice infected with *E. coli* O157:H7 or uninfected, treated with IgG or untreated, are summarized in **Table 1** and presented in **Figure 2**. A higher pathological score was only obtained in infected and untreated mice (**Figure 2A** showing intestine and **Figure 2B** showing kidney) as well as in infected and IgG-treated on day 6 but not in infected and IgG-treated on day 3 and 6 (**Figure 2C** intestine and **Figure 2D** kidney), infected and IgG-treated on day 3 (not shown) and uninfected controls (**Figure 2E** intestine and **Figure 2F** kidney). Statistical comparisons are presented in **Table 1** showing that *E. coli* O157:H7-infected and untreated mice have more profound intestinal pathology compared to infected and IgG-treated on day 3 and 6. The latter group of mice exhibited less intestinal infiltrates and less goblet cell depletion. Mice that were IgG-treated on day 3 also had less goblet cell depletion. Similarly in the kidney, *E. coli* O157:H7-infected and untreated mice displayed more severe tubular pathology compared to mice treated with IgG on day 3 and 6 or only on day 3.

**Table 1:** Intestinal and renal pathology in *E. coli* O157:H7-infected mice or uninfected mice.

Tissue	Mouse groups				
	EHEC + IgG day 3 and 6 n=10	EHEC + IgG day 3 n=5	EHEC + IgG day 6 n=5	EHEC n=9	Controls n=10
**Intestine**					
Inflammatory infiltrates^a^	1^b^ (0 - 2)*	1 (0 - 2) n.s.	2 (0 - 2) n.s.	2 (1 - 3)	0 (0 - 1)****
Goblet cell depletion	0 (0 - 1)**	0 (0 - 1)*	0 (0 - 1)*	1 (0 - 3)	0 (0)****
**Kidney**					
Tubular epithelial desquamation	0 (0 - 1)**	0 (0 - 2)*	2 (1 - 3) n.s.	2 (1 - 3)	0 (0)****

a Results assessed in blinded fashion are depicted as median and (range).

b 0: not observed; 1: mild; 2: moderate; 3: severe. Statistical comparisons were performed using Dunn’s procedure and comparing each group with the “EHEC” group, i.e. E. coli O157:H7 infected and not IgG treated. *P <0.05, **P < 0.01, ****P < 0.0001, n.s, not significant.

### Blood Urea Nitrogen in *E. coli* O157:H7 Infected Mice

**Figure 3:**
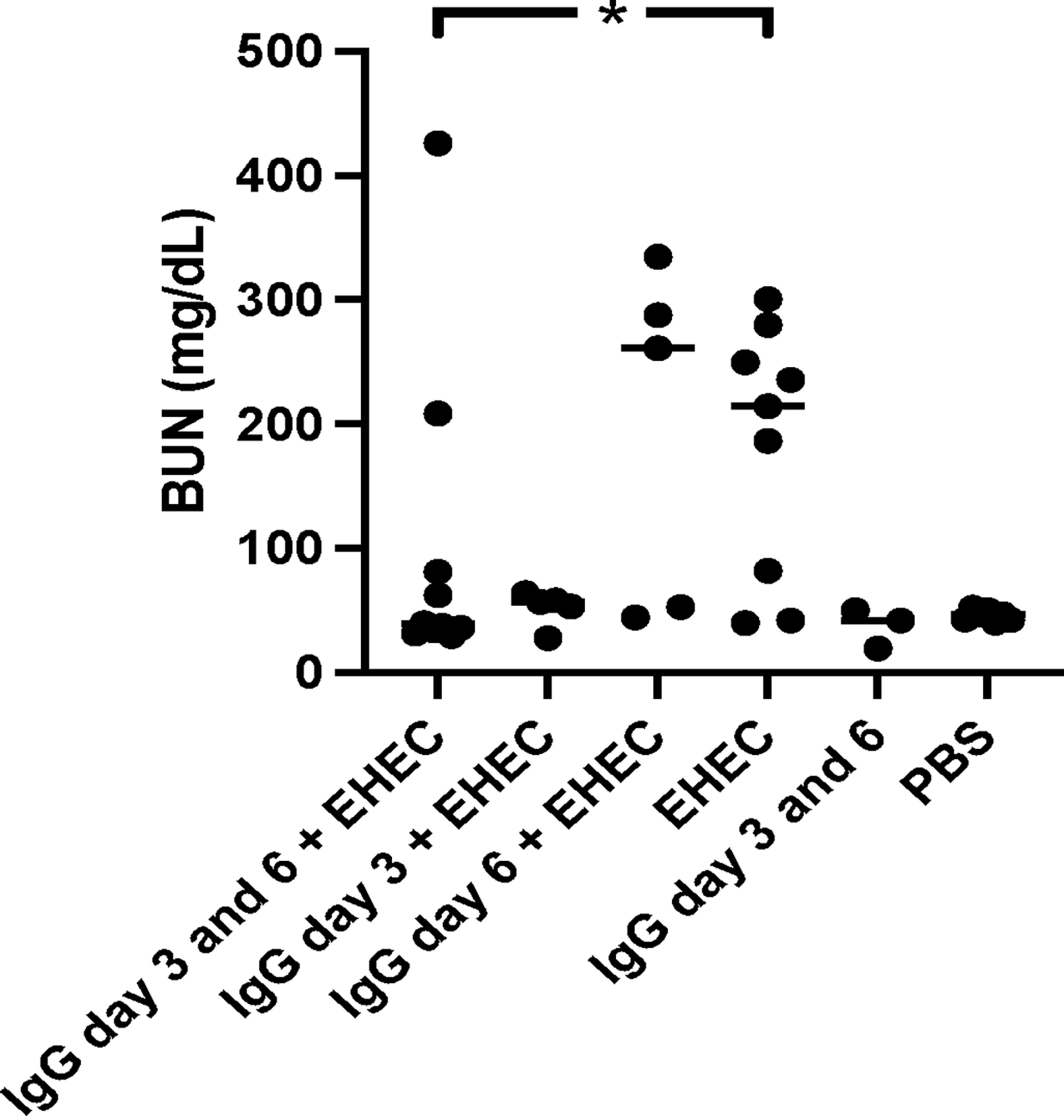
Blood urea nitrogen levels in *E. coli* O157:H7-infected mice. Blood urea nitrogen measured in plasma from *E. coli* O157:H7 (EHEC) infected and uninfected mice. Two mice in the infected and IgG-treated day 3 and 6 group developed symptoms and these are the two in this group with high BUN values. Data presented as median and individual values representing individual mice. *P < 0.05.

Blood urea nitrogen (BUN) was analyzed as a measure of kidney function in plasma taken when the mice developed symptoms or at the end of the experiment. High BUN was detected in mice that developed symptoms. A difference in BUN levels was observed between mice infected with *E. coli* O157:H7 and untreated compared to infected and treated with IgG on day 3 and 6, **Figure 3**. When comparing mice infected and IgG-treated once, on day 3 or day 6, BUN did not differ compared with mice infected with *E. coli* O157:H7 and left untreated. BUN increases upon kidney injury as well as during dehydration. As mice in the group that was infected and IgG-treated on day 3 exhibited weight loss, but did not show an increase in BUN, this could suggest that the BUN increase in the infected and untreated group, or the infected and IgG-treated on day 6, was mostly due to renal injury.

Taken together, the data show that mice infected with *E. coli* O157:H7 were well colonized, developed weight loss, clinical signs of disease, renal failure (BUN increase) as well as intestinal and renal pathology. IgG treatment on day 3 post-inoculation decreased intestinal colonization, improved survival and decreased intestinal and renal pathology. IgG treatment of infected mice on days 3 and 6 improved survival, decreased intestinal and renal pathology and protected renal function during *E. coli* O157:H7 infection. IgG treatment on day 6 post-inoculation did not have a protective effect.

### Mouse and Human IgG Bound to *E. coli* O157:H7 Proteins

**Figure 4:**
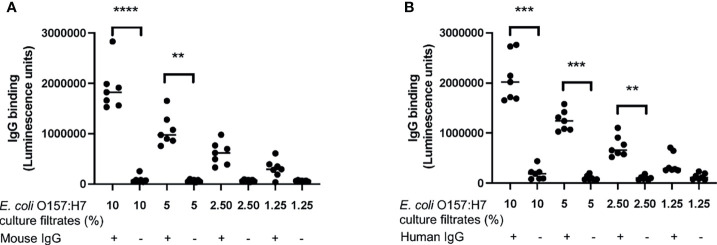
IgG binding to *E. coli* O157:H7. The binding of IgG to *E. coli* O157:H7 culture filtrate was analyzed by ELISA. **(A)** Mouse IgG exhibited dose-dependent binding to *E. coli* O157:H7 culture filtrate. **(B)** Human IgG showed dose-dependent binding to *E. coli* O157:H7 culture filtrate. The bar represents the median. **P < 0.01, ***P < 0.001, ****P < 0.0001.

The following experiments were designed to investigate if proteins/peptides released from *E. coli* O157:H7 bound to IgG. Mouse IgG bound to *E. coli* O157:H7 proteins in the bacterial culture filtrate in a dose-dependent manner (**Figure 4A**). Similar findings were demonstrated using commercially available human IgG Privigen (**Figure 4B**). Mouse and human IgG did not bind to purified Shiga toxin 2 or O157LPS (**Supplementary Figure 1**).

**Figure 5:**
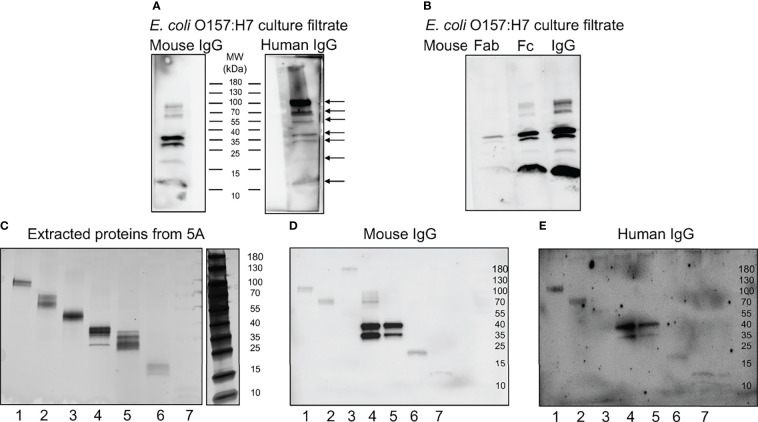
Mouse and human IgG bind specific *E*. *coli* O157:H7 proteins. *E. coli* O157:H7 proteins in culture filtrate were blotted onto membranes for detection of IgG binding. **(A)** *E. coli* O157:H7 proteins incubated with mouse IgG and human IgG. The blot detected with mouse IgG shows six bands and the blot detected with human IgG shows seven bands (arrows). Both lanes were run on the same gel. **(B)** *E. coli* O157:H7 proteins detected with mouse Fab, Fc and whole IgG showing that the binding to *E. coli* O157:H7 proteins is mediated *via* the Fc fragment. All lanes were run on the same gel and visualized by immunoblotting. **(C)** Silver stained gel of eluted proteins corresponding to the seven *E. coli* O157:H7 proteins detected with mouse and human IgG in panel **(A)**. **(D)** Immunoblot of eluted proteins [according to panel **(C)**] detected with mouse IgG. **(E)** Immunoblot of eluted proteins [according to panel **(C)**] detected with human IgG. A faint band in lane 3 was not seen in **(D)**. MW, molecular weight.

Further experimentation was carried out to investigate which proteins IgG bound to by immunoblotting. Mouse or human IgG bound to several proteins in the *E. coli* O157:H7 culture filtrate as depicted in **Figure 5A**. The protein bands that were detected using mouse IgG were also detected using human IgG (see arrows in **Figure 5A**). Six protein bands were detected with both mouse and human IgG at molecular masses of approximately 100, 60, 37, 30, 20 and 12 kDa. Using human IgG, an additional band was detected at approximately 55kDa. Similarly, when Fab and Fc fragments from mouse IgG were used to detect *E. coli* O157:H7 proteins, the Fc fragment bound to bacterial proteins showing the same pattern as full-length IgG, **Figure 5B**.

### Isolation of IgG-Binding *E. coli* O157:H7 Proteins

The proteins extracted from the different membrane segments corresponding to the seven different bands visualized by immunoblotting (**Figure 5A**) were placed on a gradient gel and detected by silver staining, **Figure 5C**. Binding of mouse and human IgG to the isolated protein bands confirmed that the correct proteins were extracted, **Figures 5D, E**, respectively.

### Identification of *E. coli* O157:H7 Proteins That Bound to Mouse and Human IgG

Extracted proteins, corresponding to the different protein band sizes binding to mouse or human IgG were analyzed by mass spectrometry. The proteins were identified based on specific criteria such as size, number of peptides, peptide score and were analyzed for distinct peptide sequences from the same protein that could be identified in more than one specimen. Proteins in segments four and five were analyzed for duplicates because similar bands were found in the extracts. Nine proteins were found in segments four and five and some of these were also identified in the other specimens as summarized in **Table 2**. EspP and translation elongation factor Tu (EF-Tu) were found in five segments each. EspP is known to be involved in bacterial virulence (14) and patients with EHEC infection mount an antibody response to EspP (28) whereas EF-Tu functions primarily intracellularly with certain membrane-associated secondary functions (29). EspP was identified as a possible target for IgG binding and studied further.

**Table 2:** Proteins that bound IgG assayed by mass spectrometry.

Extracted proteins^a^	kDa	1^b^	2^b^	3^c^	4^b^	5^b^	6^b^	7^b^
EspP	104	+	+	+	+	+		
Chaperone protein DnaK	69		+	+	+	+		
Translation elongation factor thermo unstable	43			+	+	+	+	+
NAD-dependent glyceraldehyde-3-phosphate dehydrogenase	35				+	+		
L-asparaginase	36				+	+		
Transaldolase	35				+	+		
Galactose/methyl galactoside ABC transporter, substrate-binding protein MglB	35				+	+		
Outer membrane protein A precursor	38				+	+	+	+
Outer membrane beta-barrel assembly protein BamC	36				+	+		

EspP, Extracellular serine protease P.

a Extracted proteins from E. coli O157:H7 culture filtrates bound to IgG and cut out from a membrane. Seven bands were identified corresponding to columns 1-7 (**Figure 5C**). Proteins are shown if peptide sequences from the same protein were identified in more than one column.

b Extracted proteins from E. coli O157:H7 culture filtrates detected with both mouse and human IgG.

c Extracted proteins from E. coli O157:H7 culture filtrates detected with only human IgG.

### Mouse and Human IgG Bound to Purified EspP

**Figure 6:**
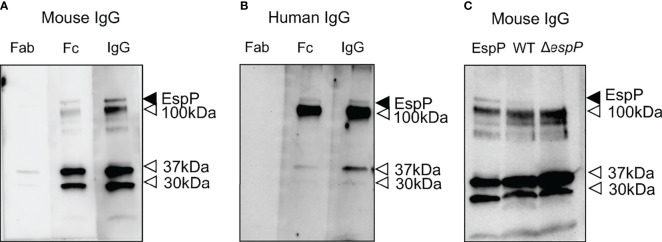
Mouse IgG and human IgG bind to EspP. Purified EspP loaded on to a membrane was analyzed for IgG binding. **(A)** Mouse whole IgG and the Fc fragment, but not the Fab fragment, bound to EspP. All lanes were run on the same gel. **(B)** Human whole IgG and the Fc fragment, but not the Fab fragment, bound to EspP. All lanes were run on the same gel. **(C)** Detection of EspP in culture filtrate from wild-type *E. coli* O157:H7 but not in the Δ*espP* strain run alongside purified EspP bound to mouse IgG. Full-length EspP detected at 104 kDa with mouse or human IgG, marked with a black arrowhead. Bands detected at different molecular weights than the full-length EspP marked with an open arrowhead representing proteins other than EspP that bind IgG.

Purified EspP was used to exhibit binding to both mouse and human IgG (**Figure 6**). Binding was mediated by the Fc domain, **Figure 6A** showing binding to mouse IgG and **Figure 6B** showing binding to human IgG. Purified EspP is expected to be visualized at 104 kDa (28). Unfortunately, we do not have a specific anti-EspP antibody. Bands were detected at 104 kDa as well as at 37, 30 and 12 kDa by both mouse and human IgG and their Fc domains.

### Binding of Mouse IgG to Culture Filtrates of Wild-Type Versus Δ*espP* *E. coli* O157:H7

Culture filtrates from wild-type and Δ*espP* *E. coli* O157:H7 were loaded onto a membrane alongside purified EspP as a control. A band at 104 kDa was detected with mouse IgG in the culture filtrate from the wild-type strain, but not in the culture filtrate from the Δ*espP* strain (**Figure 6C**). The lower bands (100, 37, 30 and 12 kDa) in **Figures 6A–C** most probably represent IgG binding to unrelated proteins as they are even visualized in the sample containing Δ*espP* *E. coli* O157:H7.

### Mouse and Human IgG Inhibit EspP-Mediated Pepsin Cleavage

**Figure 7:**
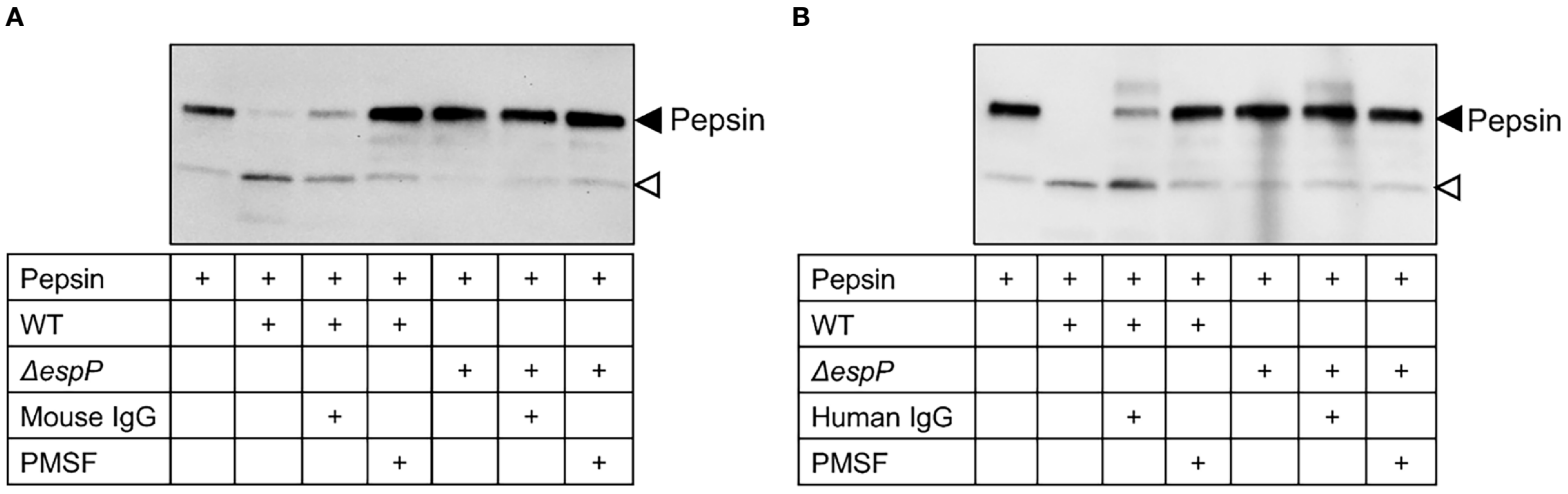
Mouse and human IgG inhibit EspP-mediated pepsin cleavage. Pepsin incubated alone or with wild-type or Δ*espP E. coli* O157:H7 culture filtrates was loaded onto a membrane for detection of pepsin cleavage. Supernatant pre-incubated with mouse IgG **(A)** or human IgG **(B)** partially inhibited EspP activity. Full-length pepsin was detected at approximately 40kDa, marked with a black arrowhead, and the cleavage product was detected at approximately 20kDa marked with an open arrowhead. WT, culture filtrate from the wild-type *E. coli* O157:H7 strain; Δ*espP*, culture filtrate from the EspP mutant; IgG, immunoglobulin G; PMSF, phenylmethylsulfonyl fluoride.

Culture filtrates from wild-type and Δ*espP* *E. coli* O157:H7 were incubated with pepsin for 16 h and the samples were transferred to a membrane. Pepsin was cleaved by EspP in wild-type culture filtrates demonstrated as a cleavage product at approximately 20 kDa. Cleavage of pepsin was not detected in Δ*espP* culture filtrates (**Figure 7**, a weak band at the location shown by the open-headed arrow is also present in the absence of bacterial culture filtrate). The EspP activity in wild-type *E. coli* O157:H7 culture filtrate was partially inhibited by mouse IgG (**Figure 7A**) and human IgG (**Figure 7B**) compared to PMSF, the positive control used for complete inhibition.

## Discussion

EHEC-associated HUS is a life-threatening condition for which there currently is no effective treatment. Here we used a mouse model of *E. coli* O157:H7 infection and show that IgG treatment improved survival, decreased intestinal and renal pathology and protected renal function. Importantly, treatment was given after inoculation, which would resemble the clinical setting in which the patient presents when infection has become symptomatic. Mouse IgG as well as commercially available human IgG were shown to bind to EspP and neutralize its catalytic activity. EspP is a very potent serine protease secreted by EHEC strains that exhibits enzymatic activity on both bacterial and host proteins and has been shown to be an important EHEC virulence factor (14). The results show that murine and human IgG bind to EspP affecting its activity and protect the intestine and kidney during *E. coli* O157:H7 infection, ultimately improving host survival.

EspP subtype alpha is specifically associated with *E. coli* O157:H7 and found in highly virulent strains (13, 14, 30). This protease plays an important role in the adherence of *E. coli* O157:H7 to bovine and human intestinal cells (12, 31) as well as in biofilm formation (31). Monomers of EspP were shown to assemble into oligomers thereby contributing to adherence, biofilm formation and HeLa cell injury (32). Exposure of colonoid cultures to EspP induced brush border damage (33), cell shedding and loss of cellular structure as well as cell death (34). A cytotoxic effect of EspP was even shown using Vero cells (35). Furthermore, EspP elicited ion transport in rat jejunal tissue (36) and human colonoid monolayers (11) indicating a role in the induction of diarrhea. EspP induced actin remodeling in human intestinal epithelial cells leading to Shiga toxin uptake by macropinocytosis (37). These studies indicate that EspP plays a major role in intestinal injury and Shiga toxin uptake during *E. coli* O157:H7 infection. This can explain how treatment with IgG, that binds to EspP, could decrease intestinal cell injury *in vivo*, and protect mice from the devastating consequences of *E. coli* O157:H7 infection, namely renal failure and death.

Not all the above-mentioned effects of EspP are associated with its catalytic activity. The cytotoxic activity is enzymatic but its effects on ion transport in colonoid cells are independent of enzymatic activity (11). EspP was also shown to have a catalytic effect on bacterial T3SS effectors, EspA, EspB and EspD, thereby regulating effector translocation into host intestinal cells (26). It was further shown to inactivate EHEC hemolysin abolishing its hemolytic activity (38). It has been proposed that EspP could thereby regulate bacterial virulence (38).

In addition to its enzymatic effects on bacterial proteins, EspP can cleave host proteins involved in coagulation and complement activation. It cleaved coagulation factor V, involved in thrombin generation. Cleavage of factor V could promote bleeding, such as during hemorrhagic colitis (28). Furthermore, EspP interacted directly with coagulation factors XII, VIII, VII, and prothrombin, decreasing their activity, which could also decrease clotting (39). Moreover, EspP was shown to cleave and inactivate complement factors C3/C3b and C5 (15), and by impairing the complement response to the infectious agent could promote bacterial pathogenesis. Thus, EspP seems to be crucial for bacterial virulence and an impaired host response. EspP also cleaves pepsin, an enzymatic activity utilized in the current study to demonstrate the inhibitory effect of IgG, and it recognizes a variety of amino acid sequences in its different substrates (40). Importantly, in order for EspP to exert an effect on coagulation and complement it would need to gain access to the systemic circulation. Patients with EHEC infection mounted an IgG response to EspP (28) suggesting that EspP could reach the circulation.

The intraperitoneal IgG treatment given in the current study would bind to EspP locally in the intestine, thereby interacting with EspP where it is secreted. We hypothesize that IgG is transported into the intestinal lumen across the epithelial cell mucosal barrier as IgG has been previously shown to undergo receptor-mediated transcytosis in epithelial cell mucosal barriers (41). Intraperitoneal administration of IgG on day 3 decreased *E. coli* O157:H7 colonization. This is in line with the effect of colostrum, that is rich in IgG and has been shown to affect intestinal attachment and colonization of *E. coli* O157:H7 in mice (42). In our model we used streptomycin to affect the intestinal microflora and effectively colonize the intestine with *E. coli* O157:H7 (24). Streptomycin would be expected to have an impact on the intestinal mucosa and we cannot rule out that it affected colonization during IgG treatment.

*E. coli* O157:H7-infected mice are prone to develop symptoms and/or lose weight during disease development, as shown in the group of mice inoculated with *E. coli* O157:H7 and left untreated, in which 8/9 developed symptoms. An indication for euthanasia in this model is the development of symptoms and/or weight loss of ≥ 20%. In the group of mice that were IgG-treated on day 3 and 6, only 2/10 developed symptoms and these mice did not lose weight. However, a certain degree of weight loss was observed in the group of mice that was IgG-treated on day 3. This weight loss was not deemed significant (did not reach 20%) and 4/5 mice were protected from the development of symptoms. Thus, even treatment on day 3 alone had a protective effect.

As EspP most probably reaches the circulation, even intravenous IgG treatment could be effective during EHEC infection, albeit possibly not as protective in the intestine. It is of importance to give the treatment early in the course of infection. Treatment on day 3 or day 3 and 6 post-inoculation with *E. coli* O157:H7 was effective in promoting mouse survival and protecting mice from intestinal and renal injury, whereas treatment on day 6 was not protective. In human infection this would suggest that IgG treatment should be investigated for treatment before the development of fulminant HUS and could be used during outbreaks of virulent EHEC strains.

Human IgG used in the present investigation was commercially available Privigen. Batches of purified IgG are pooled from multiple blood donors. Most of these donors have most probably not been infected with EHEC, or enteropathogenic *E. coli* (EPEC) that secrete a serine protease termed EspC, with homology to EspP (43). The binding of pooled human IgG to EspP was mediated by the Fc fragment. Proteins of bacterial or human origin can bind the Fc fragment of IgG, and this property is utilized for antibody purification, for example, using protein G (44). In the intestinal and bronchial mucosa Fc-binding protein Fcγbp effectively transfers IgG across the mucosal layer (45). As this is Fc, and not Fab, binding it would not entail recognition of a specific antigen. This also explains why IgG from laboratory bred mouse sera could bind to EspP by the Fc domain.

The IgG preparation used herein is available clinically for other indications. We suggest that commercially available IgG be evaluated in patients with EHEC infection. The finding that bovine colostrum containing high levels of IgG, administered orally, reduced diarrhea in patients with EHEC infection (21) supports the finding that IgG could have a protective effect if given early in the course of infection. Similar findings were demonstrated in mice given IgG-enriched bovine colostrum orally (42). Future investigations should address which route of administration is most effective, oral or intravenous, and if IgG is effective during human EHEC infection.

## Data Availability Statement

The datasets presented in this study can be found in online repositories. The names of the repository/repositories and accession number(s) can be found below: http://www.proteomexchange.org/, accession ID: PXD029563.

## Ethics Statement

The animal study was reviewed and approved by the animal ethics committee of Lund University.

## Author Contributions

AT performed experiments, analyzed the data, and wrote the paper. VS contributed conceptually and with the *E. coli* O157:H7 strain lacking *espP*. OK contributed conceptually and with the purified EspP. SL performed experiments and analyzed the data. A-CK performed experiments and analyzed the data. DK contributed to conception, designed the analysis, analyzed data and wrote the paper. IA conceived and designed the analysis, performed experiments, analyzed the data, and wrote the paper. All authors contributed to the article and approved the submitted version.

## Funding

The Swedish Research Council (2021-02200, 2017-01920 and K2015-99X-22877-01-6), The Knut and Alice Wallenberg Foundation (Wallenberg Clinical Scholar 2015.0320), Skåne Centre of Excellence in Health, The IngaBritt and Arne Lundberg’s Research Foundation, Olle Engkvist Byggmästare Foundation (all to DK). VS was supported by NIH grants: AI053067, AI154597, and AI155398. OK was supported by NIH grant P01 AI125181. SL was supported by a research fellowship from the Deutsche Forschungsgemeinschaft (LO 2021/2–1). The funding sources had no involvement in planning and carrying out any aspect of the project.

## Conflict of Interest

The authors declare that the research was conducted in the absence of any commercial or financial relationships that could be construed as a potential conflict of interest.

## Publisher’s Note

All claims expressed in this article are solely those of the authors and do not necessarily represent those of their affiliated organizations, or those of the publisher, the editors and the reviewers. Any product that may be evaluated in this article, or claim that may be made by its manufacturer, is not guaranteed or endorsed by the publisher.

## References

[1] Tarr PI Gordon CA Chandler WL. Shiga-Toxin-Producing *Escherichia coli* and Haemolytic Uraemic Syndrome. Lancet (2005) 365(9464):1073–86. doi: 10.1016/S0140-6736(05)71144-2 15781103

[2] McKee ML O'Brien AD. Investigation of Enterohemorrhagic *Escherichia coli* O157:H7 Adherence Characteristics and Invasion Potential Reveals a New Attachment Pattern Shared by Intestinal *E. Coli* . Infect Immun (1995) 63(5):2070–4. doi: 10.1128/iai.63.5.2070-2074.1995 PMC173266 7537254

[3] Karmali MA Petric M Lim C Fleming PC Arbus GS Lior H. The Association Between Idiopathic Hemolytic Uremic Syndrome and Infection by Verotoxin-Producing *Escherichia coli* . J Infect Dis (1985) 151(5):775–82. doi: 10.1093/infdis/151.5.775 3886804

[4] Békássy ZD Calderon Toledo C Leoj G Kristoffersson A Leopold SR Perez MT. Intestinal Damage in Enterohemorrhagic *Escherichia coli* Infection. Pediatr Nephrol (2011) 26(11):2059–71. doi: 10.1007/s00467-010-1616-9 20809220

[5] Mead PS Griffin PM. *Escherichia coli* O157:H7. Lancet (1998) 352(9135):1207–12. doi: 10.1016/s0140-6736(98)01267-7 9777854

[6] Jerse AE Yu J Tall BD Kaper JB. A Genetic Locus of Enteropathogenic *Escherichia coli* Necessary for the Production of Attaching and Effacing Lesions on Tissue Culture Cells. Proc Natl Acad Sci U S A (1990) 87(20):7839–43. doi: 10.1073/pnas.87.20.7839 PMC54845 2172966

[7] Kaper JB. Enterohemorrhagic *Escherichia coli* . Curr Opin Microbiol (1998) 1(1):103–8. doi: 10.1016/s1369-5274(98)80149-5 10066458

[8] Frankel G Phillips AD Rosenshine I Dougan G Kaper JB Knutton S. Enteropathogenic and Enterohaemorrhagic *Escherichia coli*: More Subversive Elements. Mol Microbiol (1998) 30(5):911–21. doi: 10.1046/j.1365-2958.1998.01144.x 9988469

[9] Newton HJ Sloan J Bulach DM Seemann T Allison CC Tauschek M. Shiga Toxin-Producing *Escherichia coli* Strains Negative for Locus of Enterocyte Effacement. Emerg Infect Dis (2009) 15(3):372–80. doi: 10.3201/eid1503.080631 PMC2681110 19239748

[10] Ruiz-Perez F Nataro JP. Bacterial Serine Proteases Secreted by the Autotransporter Pathway: Classification, Specificity, and Role in Virulence. Cell Mol Life Sci (2014) 71(5):745–70. doi: 10.1007/s00018-013-1355-8 PMC3871983 23689588

[11] Tse CM In JG Yin J Donowitz M Doucet M Foulke-Abel J. Enterohemorrhagic E. coli (EHEC)-Secreted Serine Protease EspP Stimulates Electrogenic Ion Transport in Human Colonoid Monolayers. Toxins (2018) 10(9):351. doi: 10.3390/toxins10090351 PMC6162544 30200426

[12] Dziva F Mahajan A Cameron P Currie C McKendrick IJ Wallis TS. EspP, a Type V-Secreted Serine Protease of Enterohaemorrhagic *Escherichia coli* O157:H7, Influences Intestinal Colonization of Calves and Adherence to Bovine Primary Intestinal Epithelial Cells. FEMS Microbiol Lett (2007) 271(2):258–64. doi: 10.1111/j.1574-6968.2007.00724.x 17451446

[13] Khan AB Naim A Orth D Grif K Mohsin M Prager R. Serine Protease espP Subtype Alpha, But Not Beta or Gamma, of Shiga Toxin-Producing *Escherichia coli* is Associated With Highly Pathogenic Serogroups. Int J Med Microbiol (2009) 299(4):247–54. doi: 10.1016/j.ijmm.2008.08.006 19036636

[14] Brockmeyer J Bielaszewska M Fruth A Bonn ML Mellmann A Humpf HU. Subtypes of the Plasmid-Encoded Serine Protease EspP in Shiga Toxin-Producing *Escherichia coli*: Distribution, Secretion, and Proteolytic Activity. Appl Environ Microbiol (2007) 73(20):6351–9. doi: 10.1128/aem.00920-07 PMC2075056 17704265

[15] Orth D Ehrlenbach S Brockmeyer J Khan AB Huber G Karch H. EspP, a Serine Protease of Enterohemorrhagic *Escherichia coli*, Impairs Complement Activation by Cleaving Complement Factors C3/C3b and C5. Infect Immun (2010) 78(10):4294–301. doi: 10.1128/iai.00488-10 PMC2950363 20643852

[16] Bitzan M Poole R Mehran M Sicard E Brockus C Thuning-Roberson C. Safety and Pharmacokinetics of Chimeric Anti-Shiga Toxin 1 and Anti-Shiga Toxin 2 Monoclonal Antibodies in Healthy Volunteers. Antimicrob Agents Chemother (2009) 53(7):3081–7. doi: 10.1128/aac.01661-08 PMC2704659 19414580

[17] Kamada N Sakamoto K Seo SU Zeng MY Kim YG Cascalho M. Humoral Immunity in the Gut Selectively Targets Phenotypically Virulent Attaching-and-Effacing Bacteria for Intraluminal Elimination. Cell Host Microbe (2015) 17(5):617–27. doi: 10.1016/j.chom.2015.04.001 PMC4433422 25936799

[18] Mühlen S Dersch P. Treatment Strategies for Infections With Shiga Toxin-Producing *Escherichia coli* . Front Cell Infect Microbiol (2020) 10:169. doi: 10.3389/fcimb.2020.00169 32435624 PMC7218068

[19] Amaral JA De Franco MT Zapata-Quintanilla L Carbonare SB. *In Vitro* Reactivity and Growth Inhibition of EPEC Serotype O111 and STEC Serotypes O111 and O157 by Homologous and Heterologous Chicken Egg Yolk Antibody. Vet Res Commun (2008) 32(4):281–90. doi: 10.1007/s11259-007-9029-3 18071921

[20] Ashkenazi S Cleary TG Lopez E Pickering LK. Anticytotoxin-Neutralizing Antibodies in Immune Globulin Preparations: Potential Use in Hemolytic-Uremic Syndrome. J Pediatr (1988) 113(6):1008–14. doi: 10.1016/s0022-3476(88)80572-9 3057156

[21] Huppertz HI Rutkowski S Busch DH Eisebit R Lissner R Karch H. Bovine Colostrum Ameliorates Diarrhea in Infection With Diarrheagenic *Escherichia coli*, Shiga Toxin-Producing *E. coli*, and *E. coli* Expressing Intimin and Hemolysin. J Pediatr Gastroenterol Nutr (1999) 29(4):452–6. doi: 10.1097/00005176-199910000-00015 10512407

[22] Sheth KJ Gill JC Leichter HE. High-Dose Intravenous Gamma Globulin Infusions in Hemolytic-Uremic Syndrome: A Preliminary Report. Am J Dis Child (1990) 144(3):268–70. doi: 10.1001/archpedi.1990.02150270014009 1689539

[23] Robson WL Fick GH Jadavji T Leung AK. The Use of Intravenous Gammaglobulin in the Treatment of Typical Hemolytic Uremic Syndrome. Pediatr Nephrol (1991) 5(3):289–92. doi: 10.1007/bf00867478 1714289

[24] Calderon Toledo C Rogers TJ Svensson M Tati R Fischer H Svanborg C. Shiga Toxin-Mediated Disease in MyD88-Deficient Mice Infected With *Escherichia coli* O157:H7. Am J Pathol (2008) 173(5):1428–39. doi: 10.2353/ajpath.2008.071218 PMC2570133 18832584

[25] O'Brien AD Melton AR Schmitt CK McKee ML Batts ML Griffin DE. Profile of *Escherichia coli* O157:H7 Pathogen Responsible for Hamburger-Borne Outbreak of Hemorrhagic Colitis and Hemolytic Uremic Syndrome in Washington. J Clin Microbiol (1993) 31(10):2799–801. doi: 10.1128/jcm.31.10.2799-2801.1993 PMC266020 8253989

[26] Cameron EA Curtis MM Kumar A Dunny GM Sperandio V. Microbiota and Pathogen Proteases Modulate Type III Secretion Activity in Enterohemorrhagic *Escherichia coli* . mBio (2018) 9(6):e02204–18. doi: 10.1128/mBio.02204-18 PMC6282197 30514785

[27] Szewczyk B Summers DF. Preparative Elution of Proteins Blotted to Immobilon Membranes. Anal Biochem (1988) 168(1):48–53. doi: 10.1016/0003-2697(88)90008-5 3364716

[28] Brunder W Schmidt H Karch H. EspP, a Novel Extracellular Serine Protease of Enterohaemorrhagic *Escherichia coli* O157:H7 Cleaves Human Coagulation Factor V. Mol Microbiol (1997) 24(4):767–78. doi: 10.1046/j.1365-2958.1997.3871751.x 9194704

[29] Harvey KL Jarocki VM Charles IG Djordjevic SP. The Diverse Functional Roles of Elongation Factor Tu (EF-Tu) in Microbial Pathogenesis. Front Microbiol (2019) 10:2351. doi: 10.3389/fmicb.2019.02351 31708880 PMC6822514

[30] Weiss A Brockmeyer J. Prevalence, Biogenesis, and Functionality of the Serine Protease Autotransporter EspP. Toxins (2012) 5(1):25–48. doi: 10.3390/toxins5010025 23274272 PMC3564066

[31] Puttamreddy S Cornick NA Minion FC. Genome-Wide Transposon Mutagenesis Reveals a Role for Po157 Genes in Biofilm Development in *Escherichia coli* O157:H7 Edl933. Infect Immun (2010) 78(6):2377–84. doi: 10.1128/iai.00156-10 PMC2876562 20351142

[32] Xicohtencatl-Cortes J Saldaña Z Deng W Castañeda E Freer E Tarr PI. Bacterial Macroscopic Rope-Like Fibers With Cytopathic and Adhesive Properties. J Biol Chem (2010) 285(42):32336–42. doi: 10.1074/jbc.M110.162248 PMC2952234 20688909

[33] In J Foulke-Abel J Zachos NC Hansen AM Kaper JB Bernstein HD. Enterohemorrhagic *Escherichia coli* Reduce Mucus and Intermicrovillar Bridges in Human Stem Cell-Derived Colonoids. Cell Mol Gastroenterol Hepatol (2016) 2(1):48–62.e3. doi: 10.1016/j.jcmgh.2015.10.001 26855967 PMC4740923

[34] In JG Yin J Atanga R Doucet M Cole RN DeVine L. Epithelial WNT2B and Desert Hedgehog are Necessary for Human Colonoid Regeneration After Bacterial Cytotoxin Injury. iScience (2020) 23(10):101618. doi: 10.1016/j.isci.2020.101618 33089106 PMC7559866

[35] Djafari S Ebel F Deibel C Krämer S Hudel M Chakraborty T. Characterization of an Exported Protease From Shiga Toxin-Producing *Escherichia coli* . Mol Microbiol (1997) 25(4):771–84. doi: 10.1046/j.1365-2958.1997.5141874.x 9379905

[36] Mellies JL Navarro-Garcia F Okeke I Frederickson J Nataro JP Kaper JB. espC Pathogenicity Island of Enteropathogenic *Escherichia coli* Encodes an Enterotoxin. Infect Immun (2001) 69(1):315–24. doi: 10.1128/iai.69.1.315-324.2001 PMC97886 11119520

[37] In J Lukyanenko V Foulke-Abel J Hubbard AL Delannoy M Hansen AM. Serine Protease EspP From Enterohemorrhagic *Escherichia coli* is Sufficient to Induce Shiga Toxin Macropinocytosis in Intestinal Epithelium. PloS One (2013) 8(7):e69196. doi: 10.1371/journal.pone.0069196 23874912 PMC3715455

[38] Brockmeyer J Aldick T Soltwisch J Zhang W Tarr PI Weiss A. Enterohaemorrhagic *Escherichia coli* Haemolysin is Cleaved and Inactivated by Serine Protease Esppα. Environ Microbiol (2011) 13(5):1327–41. doi: 10.1111/j.1462-2920.2011.02431.x PMC3472028 21352460

[39] Kuo KH Khan S Rand ML Mian HS Brnjac E Sandercock LE. EspP, an Extracellular Serine Protease From Enterohemorrhagic *E. coli*, Reduces Coagulation Factor Activities, Reduces Clot Strength, and Promotes Clot Lysis. PloS One (2016) 11(3):e0149830. doi: 10.1371/journal.pone.0149830 26934472 PMC4775034

[40] Dutta PR Cappello R Navarro-Garcia F Nataro JP. Functional Comparison of Serine Protease Autotransporters of Enterobacteriaceae. Infect Immun (2002) 70(12):7105–13. doi: 10.1128/IAI.70.12.7105-7113.2002 PMC133081 12438392

[41] Spiekermann GM Finn PW Ward ES Dumont J Dickinson BL Blumberg RS. Receptor-Mediated Immunoglobulin G Transport Across Mucosal Barriers in Adult Life: Functional Expression of FcRn in the Mammalian Lung. J Exp Med (2002) 196(3):303–10. doi: 10.1084/jem.20020400 PMC2193935 12163559

[42] Funatogawa K Ide T Kirikae F Saruta K Nakano M Kirikae T. Use of Immunoglobulin Enriched Bovine Colostrum Against Oral Challenge With Enterohaemorrhagic *Escherichia col*i O157:H7 in Mice. Microbiol Immunol (2002) 46(11):761–6. doi: 10.1111/j.1348-0421.2002.tb02761.x 12516772

[43] Navarro-García F Canizalez-Roman A Sui BQ Nataro JP Azamar Y. The Serine Protease Motif of EspC From Enteropathogenic *Escherichia coli* Produces Epithelial Damage by a Mechanism Different From That of Pet Toxin From Enteroaggregative *E. coli* . Infect Immun (2004) 72(6):3609–21. doi: 10.1128/iai.72.6.3609-3621.2004 PMC415714 15155671

[44] Choe W Durgannavar TA Chung SJ. Fc-Binding Ligands of Immunoglobulin G: An Overview of High Affinity Proteins and Peptides. Materials (2016) 9(12):994. doi: 10.3390/ma9120994 PMC5456964 28774114

[45] Kobayashi K Tachibana M Tsutsumi Y. Neglected Roles of IgG Fc-Binding Protein Secreted From Airway Mucin-Producing Cells in Protecting Against SARS-CoV-2 Infection. Innate Immun (2021) 27(6):423–36. doi: 10.1177/17534259211043159 PMC8504265 34521229

